# A Suspicious Hilar Mass Revealing an Uncommon Diagnosis of Pulmonary Actinomycosis in an Immunocompromised Young Female Patient: A Case Report and Literature Review

**DOI:** 10.7759/cureus.24549

**Published:** 2022-04-28

**Authors:** Mohamed Agab, Eltaib Saad, Akram Babkir, Dorota Filipiuk, Harvey Friedman

**Affiliations:** 1 Internal Medicine, AMITA Health Saint Francis Hospital, Evanston, USA; 2 Pathology, AMITA Health Saint Francis Hospital, Evanston, USA; 3 Pulmonary and Critical Care, AMITA Health Saint Francis Hospital, Evanston, USA

**Keywords:** maldi-tof, suspicious lung mass, rare diagnosis, immunocompromised patients, granulomatous disease, pulmonary actinomycosis

## Abstract

Actinomycosis is a chronic inflammatory infectious disease that can affect various organ systems. Pulmonary actinomycosis is an exceptionally uncommon clinical occurrence that yet deserves special attention, as it closely mimics a broad spectrum of infectious and neoplastic lung pathologies. The non-specific nature of its clinical features and radiological appearances makes early diagnosis quite challenging. The authors reported a 25-year-female with poorly controlled diabetes mellitus and morbid obesity who presented with a one-week history of unilateral, right-sided, pleuritic chest pain and shortness of breath. Chest imaging revealed a suspicious right hilar soft tissue mass encasing the right upper lobe bronchus with post-obstructive atelectasis. Transbronchial biopsy revealed suppurative granulomatous inflammation, and anaerobic cultures from the bronchial tissues grew *Actinomyces* species that were identified using the matrix-assisted laser desorption/ionization-time of flight (MALDI-TOF) technique. A long course of penicillin-based antibiotics was employed, and follow-up imaging revealed a satisfactory response to the antimicrobial therapy. This case demonstrates that microbiological examination is imperative to accurately diagnose the etiology of suspicious lung masses in young immunocompromised hosts. It also proves the diagnostic value of the MALDI-TOF technique in the early identification of *Actinomyces* species.

## Introduction

Actinomycosis is a slowly progressive chronic granulomatous infection caused by *Actinomyces *species, which are filamentous, branching, Gram-positive, and anaerobic or microaerophilic bacteria [1-2]. These bacteria colonized the oropharynx and gastrointestinal tract in humans [3]. The most common infections are cervicofacial and abdominopelvic actinomycosis [3]. However, most body organs can be affected, including the central nervous system, musculoskeletal, and lungs [1-4]. Pulmonary actinomycosis accounts for 15% of the total disease burden [4]. The latter disease is caused by the aspiration of oropharyngeal and gastrointestinal contents into the respiratory tract [2-3,5]. The primary pulmonary disease involves bronchioles, peribronchial tissues, and alveoli [4-5]. Nevertheless, its clinical features and radiological findings are non-specific, mimicking other infectious (atypical bacterial, mycobacteria, and fungal) and neoplastic lung diseases, and leading to delayed diagnosis [5].

Herein, the authors described an unusual case of pulmonary actinomycosis in an immunocompromised young female patient that manifested radiologically as a suspicious hilar mass simulating mycobacterial and fungal infections and neoplastic diseases. The diagnosis was established by microbiological examination of the bronchial tissues using the MALDI-TOF technique, and the targeted antibiotic therapy was employed with a reasonable response to antimicrobial therapy on serial follow-up.

## Case presentation

A 25-year-old African American female presented to our emergency department (ED) complaining of a one-week history of shortness of breath and unilateral right-sided pleuritic chest pain. These symptoms were associated with a productive cough for the last four days. The patient denied fevers or rigors, night sweating, hemoptysis, anorexia, or unintentional weight loss. The review of symptoms was pertinently negative for contributory symptoms. Her past medical history was significant for type I diabetes mellitus, stage 2 chronic kidney disease (CKD), morbid obesity, and endometrial fibroids. The patient is an active smoker (a 5-pack-year for 12 years) and a regular marijuana user, but she denied alcohol abuse.

General examination revealed a distressed patient who was afebrile (temperature 37.3C) with stable blood pressure and pulse rate. She was saturating 92% on ambient air. The patient’s body mass index was 39 kg/m². The chest examination revealed reduced breathing sounds over the right mid and lower zones with rales. The rest of the systemic examination was essentially unremarkable.

Laboratory results were remarkable for elevated white cells count (13x 100/𝜇L, reference range 4.0-11.0x100/𝜇L), elevated C-reactive protein (CRP) (44 mg/dl, reference range 0-7 g/dl), and chronic stage 3 CKD (creatinine of 2.3 mg/dl at the patient’s baseline, reference range 0.6-1.3 mg/dl). Hemoglobin A1c was 13.2% (normal range < 5.7%).

**Figure 1:**
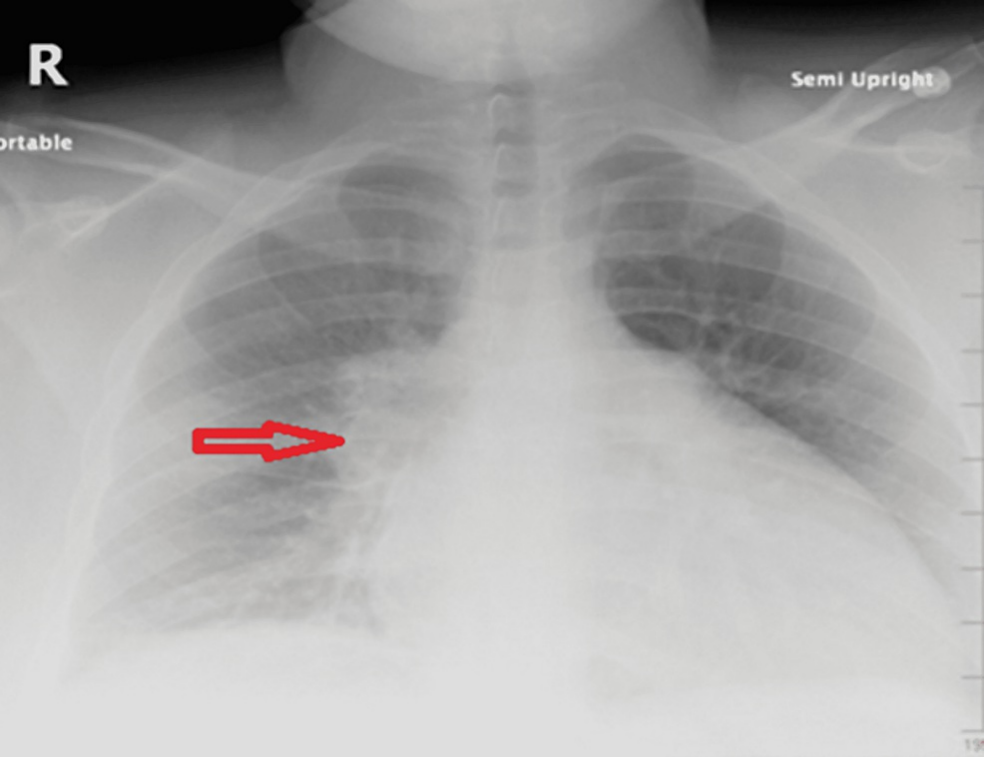
CXR showing a right hilar mass (horizontal red arrow) CXR: chest X-ray

**Figure 2:**
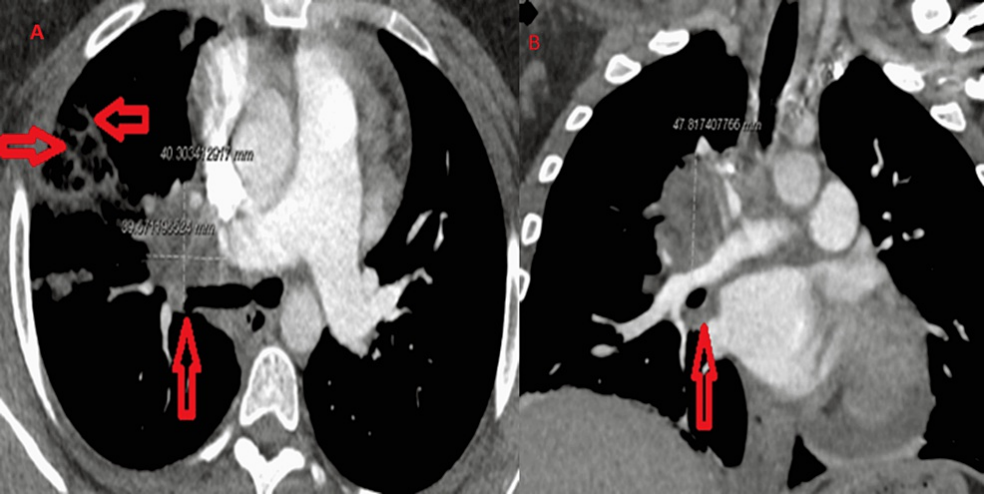
CT chest showing a right hilar mass (vertical red arrow in axial image 2A) with multifocal opacities in RUL (horizontal red arrows axial image 2A) encasing the right upper lobe bronchus and the right upper lobe’s pulmonary artery causing luminal narrowing with post-obstructive atelectasis (vertical arrow coronal image 2B) RUL: right upper lobe

Chest X-rays (CXR) revealed a right hilar mass (Figure 1). Prior imaging was not available for comparison. Contrast-enhanced computed tomography (CECT) of the chest depicted a right hilar soft tissue mass with perihilar extension, measuring about 4x 4 x 4.8 cm, encasing the right upper lobe bronchus causing luminal narrowing with post-obstructive atelectasis and right hilar adenopathy, and the mass was also encasing the pulmonary artery branches supplying the right upper lobe. Additionally, they were multifocal foci of consolidation and ground-glass opacities in the right upper lobe (RUL) extending to the subpleural space (Figures 2A-2B). There was neither mediastinal lymphadenopathy nor extra-thoracic lymphadenopathy on the abdominopelvic imaging. The differential diagnosis of these radiological appearances was broad, entailing pulmonary tuberculosis, fungal infections (histoplasmosis, coccidioidomycosis, blastomycosis, and aspergillosis), neoplastic conditions (bronchogenic carcinoma, primary or metastatic lymphoma, and sarcoma), and less likely granulomatous autoimmune diseases (necrotizing vasculitis or granulomatosis with polyangiitis).

The patient was commenced on broad-spectrum antibiotics (piperacillin-tazobactam). The cultures from the blood and sputum did not yield any growth. Nasal screening for methicillin-resistant *Staphylococcus aureus* (MRSA) was negative. Serial morning sputum samples were negative for acid-alcohol fast bacilli (AFB). Urine antigens and serology for *Histoplasma* and *Blastomyces* were also negative. Notably, the patient denied any history of recent traveling to endemic mycosis regions. In addition, Aspergillus and D-galactomannan antigens were unrevealing. Serology for human immunodeficiency virus (HIV) was unreactive. A vasculitis panel (p-ANCA, c-ANCA, anti-dsDNA, ANF, and rheumatoid factor (RF)) was negative.

Bronchoscopy with ultrasound examination showed external compression of the RUL without intraluminal lesions. No mucus, pus, or bleeding was noted. Bronchoalveolar lavage (BAL) was negative for Gram staining and AFP. Cytology from the bronchial aspirate did not reveal any malignant cells. Multiple core biopsies were obtained from the suspicious right hilar mass and were sent for histopathological and microbiological analyses. Aerobic bacterial cultures from the bronchial tissues did not grow any organisms. Additionally, fungal and mycobacterial cultures were also negative.

**Figure 3:**
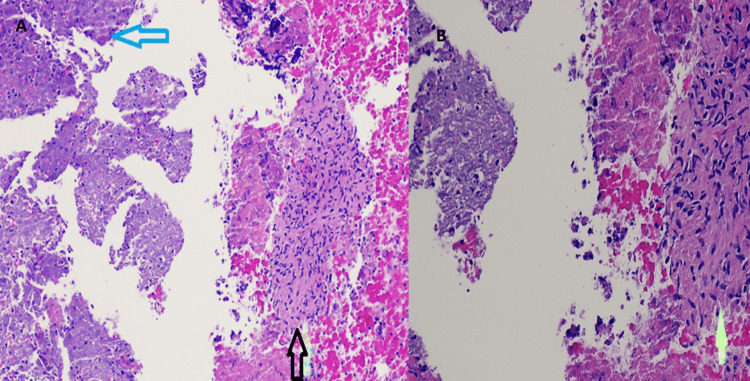
Hematoxylin & eosin stained (x40 3A and x100 3B image) of transbronchial biopsy showing suppurative granulomatous inflammatory inflammation A3) The vertical black arrow points to granuloma and the horizontal blue arrow points to necrotizing inflammation. Figure 3B) The vertical yellow arrows point to the suppurative granuloma.

Transbronchial biopsy demonstrated suppurative granulomatous inflammation (Figures 3A-3B). Staining with periodic acid-Schiff (PAS) and AFB were negative for fungi and mycobacteria, respectively. Anaerobic bacterial culture from the bronchial tissues grew *Actinomyces *species that were further identified using matrix-assisted laser desorption/ionization-time of flight (MALDI-TOF). These species were susceptible to penicillin, ceftriaxone, clindamycin, and tetracycline.

**Figure 4:**
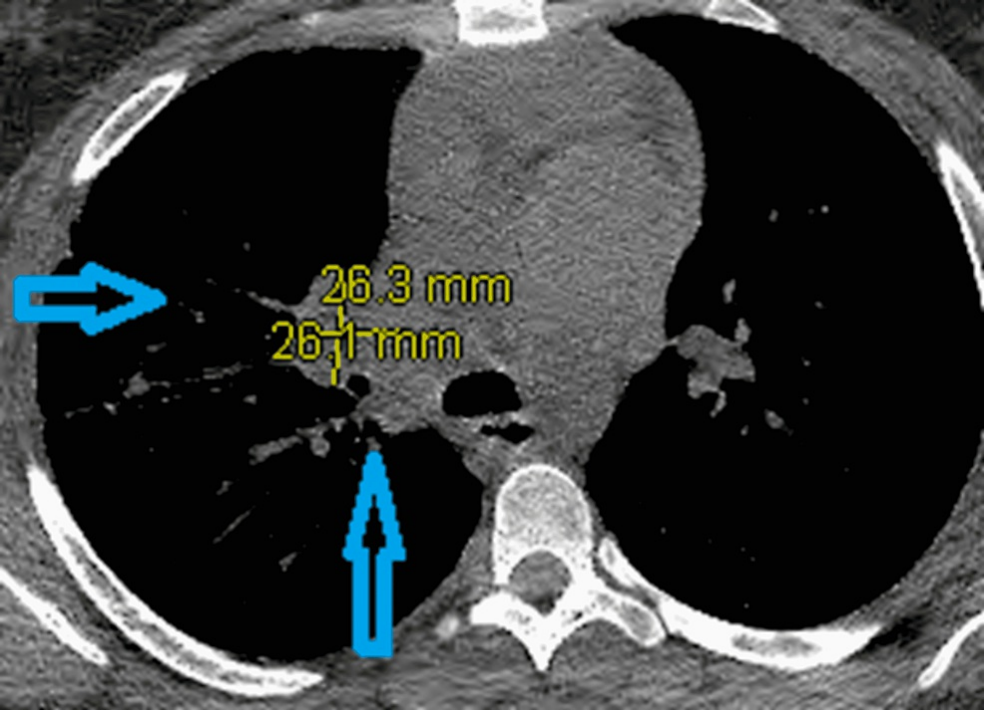
CT chest (axial image) revealed interval reduction of the right hilar mass (vertical blue arrow) with decreased RUL opacities in comparison to Figure 2A (horizontal blue arrow) RUL: right upper lobe

The patient was prescribed oral amoxicillin-clavulanate (875/125 mg, two tablets per day) for six months per the infectious disease (ID) team’s recommendation. After three months of antimicrobial therapy, a repeat CT chest scan revealed an interval reduction of the size of the right hilar mass (to 2.4 x 2.8x 2.8 cm) with decreased RUL opacities indicating a positive response to targeted antibiotics treatment (Figure 4).

## Discussion

The most-reported risk factors of pulmonary actinomycosis are poor dental hygiene, concurrent dental disease, and chronic alcoholism, which predispose to the aspiration of oropharyngeal secretions and thus serve as the principal route of lung infection [1-3]. In addition, pre-existing structural lung diseases (i.e., chronic obstructive pulmonary disease and bronchiectasis) and chronic lung infections (such as tuberculosis and aspergillosis) were associated with increased incidence of pulmonary actinomycosis by damaging lung tissues and creating an anaerobic environment that favor *Actinomyces *spp growth [4-5]. Understandably, immunocompromised hosts (diabetes mellitus, HIV, systemic chemotherapy, and solid organ transplants) are more vulnerable to encountering this disease [6-7].

Our patient had a multitude of potential risks (poorly controlled diabetes, morbid obesity, active smoking, and possibly marijuana use) that may have collectively predisposed her to the development of pulmonary actinomycosis.

The disease is more prevalent in older patients (average age was 57 years) and males (more than two-thirds, 70% of all affected patients) according to a series of 94 patients [7]. Our patient’s characteristics are not keeping with the previous series; this observation may be attributable to the patient’s immunosuppressed status that predisposed her to thoracic actinomycosis at a younger age.

The chief complaints included cough (77%), hemoptysis (65%), sputum production (60%), and less than one-third of patients had a fever, dyspnea, and chest pain [7]. Chest CT findings are quite variable, including consolidation (70%), followed by hilar lymphadenopathy (29%), atelectasis (28%), cavitation (23%), and ground-glass opacities (14%) [7]. Nevertheless, these radiological appearances are non-specific, closely resembling various infectious and neoplastic lung pathologies [2,5,7]. Hence most cases were initially misdiagnosed as lung cancer, pneumonia, or mycobacterial infection (Table 1) [7].

**Table 1:** Initial radiological diagnoses of pulmonary actinomycosis in a series of 94 patients

Initial diagnosis	Number (%)
Lung cancer	33 (35.1%)
Pneumonia	18 (19.1%)
Tuberculosis or non-tuberculous mycobacteria (NTM)	16 (17%)
Aspergillosis	8 (8.5%)
Actinomycosis	6 (6.4%)
Lung abscess	5 (5.3%)
Empyema	3 (3.2%)
Broncholithiasis	2 (2.1%)
Granuloma	2 (2.1%)
Fibrothorax	1 (1.0%)

The definitive diagnosis of pulmonary actinomycosis is principally based on histopathological and microbiological examinations of lung tissue biopsies obtained through minimally invasive approaches (CT-guided transthoracic needle biopsy), or semi-invasive (bronchoscopy-guided biopsy), and even invasive techniques (excisional open surgical biopsies) [8-9].

Histopathologic findings are characterized by chronic exudative inflammation and fibroblast proliferation [1-2,5,7]. Radiating filamentous colonies that can be visualized under hematoxylin and eosin stains (H&E) represent the gold standard for identifying *Actinomyces *spp [1-2,5,7].

It is interesting to note the low diagnostic yield of conventional bacterial culturing in isolation of *Actinomyces *spp (culture rate less than 50%) [10]. This poor sensitivity may be explained by the fastidious growth of *Actinomyces *spp, overgrowth of other bacteria even when the clinical suspicion of actinomycosis is high, and pretreatment with empirical antibiotics [10]. The MALDI-TOF technique employed in our patient allowed the early identification of *Actinomyces *spp in keeping with the recent literature that demonstrated the value of this new technique in diagnosing pulmonary actinomycosis [11]. The latter tool has been used recently for the identification of bacteria, mycobacteria, and fungi with high diagnostic reliability and accuracy [12]. It uses a laser to disperse and ionize the analyte into different molecules, which move through a vacuum, driven by an electric field, before reaching a detector membrane. The specific time-of-flight data are assembled, resulting in specific spectra that are compared to a specific database, which allows for the rapid identification of the infectious agent [12]. Furthermore, molecular techniques with polymerase chain reaction (PCR) and gene sequencing of 16S rRNA can also allow rapid and accurate identification of *Actinomyces *spp, and thus early treatment can be instituted [13-14].

Fortunately, antimicrobial therapy of pulmonary actinomycosis is highly effective, and early diagnosis is more likely to lead to a cure [5,7]. Parenteral penicillin for two to six weeks, followed by oral penicillin-based antibiotics for four to 12 months, is the treatment of choice [5,7]. Erythromycin, clindamycin, and tetracycline are alternatives for penicillin-allergic patients [5,7].

The reported patient demonstrated a reasonable response to antibiotics therapy evidenced by the follow-up imaging. The lack of clinical and radiological response to antimicrobial treatment at one month is a poor prognostic factor [9]. Surgical intervention is reserved for complicated diseases such as massive hemoptysis and pleural empyema [7].

Learning points

Pulmonary actinomycosis is an exceptionally uncommon clinical occurrence that deserves special attention. It closely mimics a broad spectrum of infectious and neoplastic lung pathologies owing to the non-specificity of its clinical and radiological features. The microbiological examination is imperative to accurately diagnose the etiology of suspicious lung masses in young immunocompromised hosts. The MALDI-TOF technique can be helpful in the rapid identification of *Actinomyces *species, allowing early diagnosis and prompt treatment, which is associated with a better outcome.

## Conclusions

Pulmonary actinomycosis is an exceptionally uncommon clinical occurrence that deserves special attention, as it closely mimics a broad spectrum of infectious and neoplastic lung diseases owing to the non-specific nature of its clinical features and radiological findings. This case demonstrates that microbiological examination is imperative to accurately diagnose the etiology of suspicious lung masses in young immunocompromised hosts. It also proves the diagnostic value of the MALDI-TOF technique in the early identification of *Actinomyces *species.
